# Medicine, Pathology and Therapeutics

**Published:** 1898-04

**Authors:** Albert T. Lytle

**Affiliations:** Buffalo, N. Y.


### Progress in Medical Science.

### MEDICINE, PATHOLOGY AND THERAPEUTICS.

CONDUCTED BY ALBERT T. LYTLE, M. D., Buffalo, N. Y.

### INTESTINAL PERISTALSIS BY ANIMAL EXTRACTS AND THE LIKE.

OTT, (*Med. Bulletin*) in experiments on intestinal peristalsis in animals, found that adrenal extract enlarged the lumen of the intestine and increased peristalsis; that spleenic extract produced marked increase of peristaltic waves; that removal of a spleen diminished peristalsis and that feeding dried spleen in the same case caused quickened peristalsis. Thymus and pituitary extracts were negative. Pancreatic extract lowered peristalsis, as do brain and thyroid. Eserin narrows lumen intestine, while pilocarpin and nicotin increase peristalsis. Irritation vagus caused marked contraction, while that of the splanchnics caused marked slowing.

### ENTEROPTOSIS.

LANGERHANS (in *Archives für Verdauungskrankheiten*, *Bd. III.*, *Heft 3*,) concludes that a moderate degree of enteroptosis, when it is consecutive upon frequent child bearing, is physiological and productive of no symptoms. The most frequent symptoms of this condition are those of aggravated dyspepsia, pain and demonstrable abnormality of secretory and motor functions. The descent of abdominal organs changes the mechanical relations, so that lasting injuries sometimes result, due to pressure upon the unprotected kidney, to increased demand upon the propulsive muscles of the stomach and always to a stretching of the mesentery. By the cumulative effect of these numerous and continuous irritations, even though each in itself may be slight, the central nervous system becomes exhausted and, according to individual predisposition, neurasthenia or hysteria with especially pronounced abdominal symptoms supervenes. Chronic constipation is generally a marked condition. Treatment consists—first and most important—in gymnastics of the abdominal muscles and then symptomatic therapeutics.—*Boston Med. and Surg. Jour.*

### TREATMENT OF BURNS.

KEEN and DaCosta (*Practitioner*) claim excellent results from the following use of normal salt solution (one teaspoonful table salt to the pint of water) : The injured part is wrapped in absorbent cotton, soaked with normal salt solution and a bandage applied which contains a trap door for the frequent addition of the solution and the part put strictly at rest. They claim that pain is quickly allayed and that granulation is very rapid, while if skin grafts are required the raw surface is prepared. Pozzi uses saturated solution of potassium nitrate in the same way and claims rapid abolishment of pain. Haas gives the following recipe :

| | | | |
|---|---|---|---|
| ℞ | Aristol | grs. | 75 to 150 |
| | Olive oil | " | 300 |
| | Lanolin. | | |
| | Vaselin ..... āā | " | 600 |

M. Sig.—Use as external application.

---

### INTERNAL TREATMENT OF PYELITIS.

Dr. M. A. Robin (*Deut. Med. Ztg.*, XVIII.,) advises hygienic and dietetic measures ; care to prevent chilling of the body ; strict milk diet as a basis of nourishment, all fish, chicken, veal, beans and spices to be avoided ; alcohol to be positively forbidden ; the administration of sodium benzoate internally, in amount one to four grammes per diem, to check fermentation and the deposit of uric acid. In acute and painful cases he advises a pill, as follows, to be given three times a day :

| | | | |
|---|---|---|---|
| ℞ | Larch turpentine | grs. | 90 (6.00) |
| | Camphor | " | 90 (6.00) |
| | Tr. opium | m. | 4 (0.25) |
| | " aconite | " | 3 (0.20) |

M.—In pill No. 60. div.

—*Am. Med.-Surg. Bull.*

---

### CALOMEL TO ABORT INFLUENZA.

Friedenthal (*Ther. Mont.*) believes calomel very useful in the early stages of influenza. He gives a dose amounting to two grains twice a day to adults or one grain every three or four hours. He gets a fall of temperature in a few hours with a disappearance of the neural pains and an increase in the appetite. As the treatment is inoffensive it can be generally employed. He believes that a cure can be produced in about three days.—*Ther. Gaz.*

### METHYLENE BLUE IN ATAXIC PAINS.

Lemoine (*Jour. des Prac.; Ther. Gaz.*) finds that methylene blue relieves ataxic pains ; those which seem best relieved are the darting ones of the limbs and the sensation of a tight band about the patient (girdle pains). The relief is great and prolonged. The effect comes on rapidly. The urine is colored blue in two or three hours. (Do not mistake for methyl blue or pyoktanin, which is poisonous.) The dose is from grains one to three, three or four times a day.

---

### INSOMNIA.

McPhedram (*Brit. Med. Jour.*) in the treatment of insomnia strongly urges that one seek out diligently and treat intelligently such obvious causes as may be present as disturbances of the alimentary tract, improper diet and constipation ; to stimulate the emunctories to produce free elimination of the products of metabolism ; to seek and correct local and peripheral irritations and reflex disturbances ; to stop overwork and to keep the duties of the patient well within the limits of his capacity ; to relieve anxiety; to study and improve the circulation ; to correct improper sanitation, and to inculcate good habits. Having done all these then resort to use of hypnotics if required. Attention is directed to the more than occasional serious and even fatal results from the administration of such supposedly harmless drugs as chloral, sulphonal, trional and paraldehyde, much of which is due to choked avenues of elimination, to excessive dosage and to long administration. He, of course, deprecates the use of such hypnotics as morphia, cocaine, bromides, etc.

---

### TO REMOVE SUPERFLUOUS HAIR.

| | | |
|---|---|---|
| ℞ | Tinct. iodin........................ | 3 parts. |
| | Oil Terebinthinæ.................... | 6 " |
| | Oil ricini ......................... | 8 " |
| | Spirits rect........................ | 48 " |
| | Collodion .......................... | 100 " |

M.—Paint affected part once daily for three or four successive days. When scab comes off hairs will be on under surface.

—Putte.

### FOR SOFT CORNS.

| | | | |
|---|---|---|---|
| ℞ Iodin | 0.13 gram | | gr. ii. |
| Flexible collodion | 12.00 | " | ʒ iij. |
| Alcohol | 4.00 | " | ʒ i. |
| Potassium iodid | 0.13 | " | gr. ij. |

M.—Paint on at night.

—*Am. Med.-Surg. Bull.*

---

### ANTINEURALGIC LINIMENT.

| | |
|---|---|
| ℞ Ichthyol | 1 part. |
| Mercury ointment | 1 " |
| Chloroform | 6 " |
| Spts. camphor | 6 " |

M.—Externally. Shake well. Rub upon affected part.

—*Med. Weekly.*

---

### BRONCHITIS.

| | |
|---|---|
| ℞ Terpinol. | |
| Sodium benzoate | āā grs. ii. |
| Saccharum lactis | q. s. |

M.—In pill No. 1. Make 100.
Sig.—6 to 12 per diem.

—*Prescription.*

---

### ACUTE CORYZA, IF COMMENCED EARLY.

| | |
|---|---|
| ℞ Salol | gr. xv. |
| Acid salicylic | " iij. |
| Acid tannic | " j. |
| Pulv. ac. boracid | ʒ i. |

M. et sig.—Take a pinch as a snuff every hour for six or eight hours. Beware too long use, as will cause margins of nose to become sore.

---

### ORTHOFORM.

In the *Lancet* the new substance orthoform is described as a methylic ether of amidobenzoic acid and as possessing remarkable anesthetic and analgesic properties when locally applied. It is a white crystalline powder without taste or smell and but slowly soluble in water. It is nonpoisonous, rather slow in action, but persistent in effect. It is antiseptic, diminishes secretion and can be used internally and upon broken surfaces in large amounts ; as much as 700 grains has been applied to a carcinoma of the face in one week without untoward effects. The hydrochlorate is readily soluble in water, but the solution is too acid for use in the eyes, although it can be used internally; a saturated watery solution is suitable for local use; while a dose of 7½ grains of the hydrochlorate internally several times a day produces no untoward symptoms ; it would seem indicated in gastric ulcer and cancer. Orthoform is shown to be superior in its application to cocaine or eucaine and altogether harmless so far as reported.

---

### DIPHTHERIA.

REISMAN in the *Phila. Med. Jour.* reports a case of diphtheria under peculiar conditions. A bacteriologist accidentally received some of a virulent diphtheria-germ culture into the back of his mouth ; he immediately ejected the material, but took no other precaution against infection. Two days later white patches were discovered upon the tonsils which a culture showed to be diphtheritic. Immediately 2000 antitoxin units were injected, about eight hours later another 2000 units were used, while the next day a third 2000 units were injected. The patches disappeared upon the fifth day, but despite local applications the bacillus remained for nearly three weeks. The case shows the minimum time of incubation and the curative power and necessity for large and early dosage of antitoxin.

---

### TREATMENT OF POLYNEURITIS.

THE treatment (*Med. Standard*) is divided into five groups, according to the prominent groups of symptoms : (1) Combat pain by use of morphin, antipyrin, sodium salicylate, salophen, or methylene blue internally ; by external applications of solution carbolic acid, 4 to 5 per cent. ; by judicious use of water and by hypodermic injection at painful areas of a mixture of a 2 per cent. solution acid carbolic, with a 1 per cent. solution morphine in quantity of one cubic centimeter or less. (2) Combat insomnia by use of potassium bromide, morphine (contraindicated in vagus involvement), chloralose, sulfonal, trional, paraldehyde and chloral (in alcoholic neuritis with care). (3) Combat paralysis—if craniobulbar, energetic and prompt measures are demanded—by use of hypodermic injections of caffein, ether and camphor, using faradism if dyspnea and cyanosis be present; use mustard applications, thrashing with cold cloths, faradisation, oxygen and strychnin. (4) Combat deformities due to defective attitudes and tendon retractions by the use of splints and bandages to retain the parts in normal attitudes. (5) Curative: give a diet rich in phosphorus, hot baths, salt baths, strychnin, electricity—galvanic and faradic,—fresh air and sunlight.

